# Potential Fiscal Consequences of Universal Screening for Group-B Streptococci

**DOI:** 10.1155/S1064744996000397

**Published:** 1996

**Authors:** Gilles R. G. Monif

**Affiliations:** Creighton University School of Medicine USA


Infectious Diseases in Obstetrics and Gynecology 4:211–213 (1996)
© 1997 Wiley-Liss, Inc.

### Potential Fiscal Consequences of Universal Screening for Group-B Streptococci

The proposed recommendation for extensive screening for group-B streptococci (GBS) during pregnancy published in the *Federal Register*[1] and advocated by the American Academy of Pediatrics[2] will have an impact on health-care economics to varying extents in at least 2 areas. Implementation of the pending mandate for universal screening will 1) require direct funding to expand the current proactive prenatal infectious-disease screening and 2) indirectly fuel the fiscal consequences of failure.

The cost efficiency of universal screening for GBS vs. situationally dictated prophylaxis has been approximated. The CDC cited the analysis of Stray-Pederson[3] in indicating that the cost per case prevention was <$35,000. The aggregate studies of Boyer and Gotoff[4] as well as that of Strickland et al.[5] and Yancey and Duff[6] similarly support this contention. Based on the high cost of caring for neonates with early-onset disease ($22,000–33,800), the policy of screening and treatment has been projected to be economically justified.[1,6]

Gibbs et al.[7] reviewed the 2-year experience of a universal screening program with strict application of criteria and found the compliance rate in administering indicated prophylaxis with a complex algorithm to be only 80.3%. The incidence of GBS sepsis in the last year's protocol was 0.5 per 1,000 live births. Of the 5 cases of early-onset neonatal GBS, 4 were due to a failure to identify GBS with the initial screening culture. All 3 of the 4 cases were said to be protocol violations attributed to nonselective media. The majority of hospital laboratories do not use selective media in screening for GBS. The reason is simply that, because of the additional steps required to process a specimen for GBS, the use of selected media markedly increases the cost of culturing. Had selective broth media been used, there is no absolute guarantee that the cultures would have been positive for GBS.

The recommendations for universal screening as advocated by the American Academy of Pediatrics[2] are predicated upon the desire to bring the number of cases of early- and late-onset GBS neonatal disease to zero. The proposal for universal screening deviates significantly from the prior recommendations of Minkoff and Mead[8] and the more formal policy advocated in 1992 by the American College of Obstetricians and Gynecologists (ACOG).[9] What would be the difference in terms of serious residual sequelae or death as a result of GBS neonatal disease between universal culturing and selective prophylaxis vs. selective prophylaxis? The projected numbers are exceedingly small, and the difference in cost is significant.

In June 1996, the ACOG Committee on Obstetric Practice[10] published a positional paper dealing with the prevention of early-onset GBS disease in newborns. Noting that there were no, nor were there likely to be, clinical studies comparing the efficiency of the strategies using antepartum culture and selected intrapartum prophylaxis with intrapartum prophylaxis based on clinical risk factors, the committee recommended that obstetric providers adopt 1 of 2 strategies for the prevention of early-onset GBS disease in the newborns: a strategy based on late prenatal culture (35–37 weeks) as the primary risk determinant or a strategy based solely on clinical risk factor. If the culture-based strategy is used, intrapartum antibiotic prophylaxis needs to be extended

Received July 9, 1996
Accepted July 10, 1996

Editorial

TABLE 1. Funding of proactive infectious disease screening[a]

| | Funding sources | | | | |
|---|---|---|---|---|---|
| Organism | Medicare | NE state | HMO | Blue Cross/Blue Shield | For-profit hospital |
| *Neisseria gonorrhoeae* DNA probe and *Chlamydia trichomais* (87178) | $14.67 | $23.62 | $30.80 | $12.00 | $30.00 |
| Asymptomatic bacteriuria (87086) | $11.26 | $11.91 | $22.00 | $24.00 | $21.00 |
| *Treponema pallidum* (VDRL/FTA) (86781) | $7.67 | $7.67 | —[b] | $12.00 | $33.95 |
| HBV (86289) | $17.06 | $17.45 | $17.60 | —[b] | $21.25 |
| HCV (86302 and 86303) | $20.20 | RNE | $18.70 | —[b] | $32.00 |
| HIV-1/HIV-2 (86311) | $8.67 | $27.46 | $14.30 | $20.00 | $22.00 |
| Rubella (86762) | $20.30 | $21.37 | —[b] | —[b] | $16.00 |
| GBS (82951) | $18.22 | $20.04 | $32.00 | $32.00 | $21.00 |
| *M. tuberculosis* (86585 and 86580) | Not listed | | $7.15 | $11.00 | $21.00 |
| Total | $118.05 | $129.52 | $142.55 | $111.00 | $218.20 |

[a]VDRL = venereal disease research laboratory test; FTA = fluorescent treponemal antibody testing; HBV = hepatitis B virus; HCV = hepatitis C virus; HIV-1/2 = human immunodeficiency virus types 1 and 2; GBS = group B streptococcus. Numbers in parentheses refer to billing codes.
[b]Funded within the global prenatal fee.

to all women who have positive cultures. Irrespective of which strategy is chosen, neither will prevent all early-onset GBS disease.

If you spend health-care dollars, you have to be sure the recipient receives the maximum value for the money. To save 1 infant, others may have to be compromised. A small incremental reduction in the incidence of GBS neonatal disease can probably be achieved over prophylactic therapy of high-risk categories, but at what price? The cost of screening and serial monitoring of GBS carriers and their sexual consorts will take dollars from the health-care system which may be better spend proactively on other aspects of in utero medicine, such as surveillance for hepatitis C virus, *Mycobacterium tuberculosis*, or vaginal bacteriosis.

### DIRECT FISCAL CONSEQUENCES

The infectious organisms currently screened for within the context of prenatal care are listed in Table 1; however, not all of these are funded by add-on health-care dollars. The proposed mandate for universal or comprehensive screening brings up the ugly question, "Who is going to pay for serial screening of gravidas with GBS?" Screening entails at least 2 culture sites (Vaginal and rectal) and, in some situations, 2 maternal sets of cultures. Is the cost of proactive health care of infectious-disease surveillance to grow or are tests to be deleted from proactive health care to accommodate GBS testing? Do we have the fiscal resources to do everything? Hopefully, the anser is "yes." Historically, health-maintenance organizations (HMOs) and the Federal Government pay greater lip service than actual implementation of cost for health care maintenance.

### INDIRECT FISCAL CONSEQUENCES

Irrespective of whether a policy of universal screening, selective high-risk category screening, or prophylactic high-risk categories therapy is implemented, a rare case of GBS will occur. The implied assumption that zero occurrence will be the consequence of universal screening will make ensuing cases of early-onset GBS disease a medical or legal nightmare. In a realistic sense, one has to try to imagine a family practitioner in a rural setting who had adhered to this proactive health-care mandate and failed. Under cross examination concerning a prior negative maternal culture, the physician would be asked to answer questions such as, "What quality controls are in place at the testing facilities? Were the cultures properly taken? Was selective media used? How many sites were individually sampled? Were the cultures properly handled in transport? Were all appropriate culture sites tested?" Anything short of a Lexis response to these types of questions could result in a 7-figure judgment.

### A DIFFERENT PERSPECTIVE

A plea for financial prudence should not be the essence of policy. The real problem with universal screening is that it was developed with a myopic focus. Early-onset disease caused by GBS is a major component within a larger problem, namely perinatal septicemia. Perinatal septicemia is defined as sepsis within the first 24–36 h of life.[10] The factors which select for perinatal septicemia are markedly different from those responses for neonatal septicemia. The Enterobacteriaeae are as important as GBS as the etiologic agent for serious sepsis in the first 24–36 h of life. The preventative measures for GBS should equally address the need to circumvent neonatal disease caused by the Enterobacteriaceae. The classic case of perinatal Enterobacteriaceae septicemia is a neonate born to a gravida who experiences prolonged rupture of the fetal membrane (ROM) or preterm labor and ROM. Nonprophylaxis or prophylaxis with just penicillin in a gravida with prolonged ROM or preterm labor because of the absence of GBS in the vaginal or rectal bacterial flora leaves this group at significant risk for perinatal septicemia due to, e.g., Enterobacteriaceae or *Hemophilias influenzae*.[11]

A policy to aggressively cirumvent the tragic morbidity and mortality due to early-onset GBS needs to be formulated, but its methodology must be global, not myopic.

*Gilles R.G. Monif, MD*
*Director of Infectious Diseases—OB/GYN*
*Creighton University School of Medicine*

### REFERENCES

1. Centers for Disease Control and Prevention: Prevention of group B streptococcal disease: A public health perspective. Fed Regist 59:64764–64770, 1994.
2. American Academy of Pediatrics, Committee on Infectious Diseases and Committee on Fetus and Newborn: Guidelines for prevention of group B streptococcal (GBS) infection by chemoprophylaxis. Pediatrics 90:775–778, 1992.
3. Stay-Pederson B: Economic evaluation of maternal screening to prevent congenital syphilis. Sex Transm Dis 10:167–172, 1983.
4. Boyer KM, Gotoff SP: Prevention of early-onset neonatal GBS disease with selective intrapartum chemoprophylaxis. N Engl J Med 314:1665–1668, 1986.
5. Strickland DM, Yeomans ER, Hankins GDV: Cost effectiveness of intrapartum screening and treatment for maternal group B streptococci colonization. Am J Obstet Gynecol 163:4–8, 1990.
6. Yancey MK, Duff P: An analysis of the cost-effectiveness of selected protocols for the prevention of neonatal group B streptococcal infection. Obstet Gynecol 83:367–371, 1994.
7. Gibbs RS, McDuffie RS, McNabb F, Fryer GE, Miyoshi T, Merenstein G: Neonatal group B streptococcal sepsis during 2 years of a universal screening program. Obstet Gynecol 84:496–500, 1994.
8. Minkoff H, Mead P: An obstetric approach to the prevention of early-onset group B β-hemolytic streptococcal sepsis. Am J Obstet Gynecol 154:973–977, 1986.
9. American College of Obstetricians and Gynecologists: Group B Streptococcal Infections in Pregnancy. ACOG Technical Bulletin No. 170. Washington, DC: ACOG, 1992.
10. ACOG Committee Opinion: Prevention of Early-Onset Group B Streptococcal Disease in Newborns. No. 173, June 1996.
11. Centers for Disease Control and Prevention: Prevention of perinatal group B streptococcal disease: A public health perspective. MMWR 45(RR-7):1–24, 1996.
